# 

**Published:** 1915-04-24

**Authors:** 


### Chronic Collargol Poisoning.

In a previous issue the risk was pointed out in these columns of poisoning following the too enthusiastic use of colloid metals, the latest fad in therapeutics. One swallow does not, of course, make a summer; but all the same a case of chronic silver poisoning reported in the *Journal of the American Medical Association* is instructive. Dr. Crispin was asked to operate on a young woman for supposed gall stones; she was the subject of a peculiar dark pigmentation which was diagnosed as jaundice. Examination failed to reveal any evidence of gall-bladder trouble, though a chronic appendicitis was thought to be likely. It then turned out that the patient had, on the advice of a doctor, taken collargol (colloid silver) for four years. The pigmentation was thus ascribable to chronic silver poisoning. Potassium iodide was tried as a remedy for this, but it set up acute coryza. To cure the latter hexamethylenamine was ordered, and immediately the colour began to fade. An operation was then performed, and a chronically inflamed appendix removed: the muscles and intestines were noticed to be pigmented.

### Medical Appointments.

The Council of the Royal Eye Hospital have appointed the undermentioned as temporary honorary assistant surgeons for the period of the war: F. D. Bennett, M.R.C.S.; A. E. A. Loosely, M.A., B.Ch., F.R.C.S., M.R.C.S., L.R.C.P.; and T. W. Letchworth, M.A., B.Ch., F.R.C.S., M.R.C.S., L.R.C.P. See "Round the World," page 88.)

### Gifts and Bequests.

Mr. James Senior, of Huddersfield, Yorks, brewer, has left £2,000 to the Huddersfield Royal Infirmary.

Sir George Pragnell's special appeal to the textile trade on behalf of the wounded has closed with a grand total of £21,328 9s. 11d., and the joint Red Cross and St. John Committee has tendered warm thanks to 834 subscribers.

Mr. James Hamilton Dunn, of Castlehead, Paisley, writer, of the firm of Messrs. J. A. Dunn and Allison, of Paisley, has left £1,000 each to the Royal Alexandra Infirmary and the Gleniffer Home for Incurables, and £500 each to five other Paisley charities.

Mr. Richard John Bowerman, of Pembridge Square, Bayswater, W., who left estate of the gross value of £235,025, has bequeathed the residue of his property upon trust for his wife during widowhood, and, subject thereto, £500 each to the London Hospital and the Bridewell and Bethlem Hospital.

The Samaritan Free Hospital for Women, Marylebone Road, N.W., has received from the executor of the late Miss S. A. Oxtoby, of 39 Regent's Park Road, N.W., and Harrogate, the sum of £300 from the residue left by her for distribution to charities at the discretion of her trustees. The executor, Mr. Herbert J. Jeffrey, of Bradford, stipulates that the sum shall be placed to the hospital's investment account.

### Personalia.

### BRADFORD ROYAL INFIRMARY AND ARMY SERVICE.

In addition to the withdrawal of several resident medical officers for Army service, the Bradford Royal Infirmary has lost the services temporarily of its two honorary assistant surgeons. Mr. F. W. Goyder, B.A., B.C., M.B. Cantab., F.R.C.S., was invited to take the post as one of the surgeons to a Red Cross Hospital, and left for France early in March. Mr. Peter McEwan, M.A., M.B., Ch.B., F.R.C.S. Edin., has been appointed to the staff of a casualty clearing hospital, and has proceeded to Southampton to join his unit.

### BOVRIL PICTURES.

The public is by this time aware of the picture scheme which Bovril, Limited, has initiated in connection with its business. In the present year the pictures, of which reproductions are obtainable under the firm's conditions, are called "A Private Rehearsal," by Arthur J. Elsley; "A Welsh Valley," by A. de Breanski, jun.; and "Good-Night," by Arthur J. Elsley. The titles convey the subject-matter, and those desiring proofs or gravures for the decoration of their houses or hospitals should study the Bovril picture scheme, particulars of which are readily obtainable, or may be applied for at the head offices, 152-166 Old Street, London, E.C.

### The Book Market.

LIST OF NEW BOOKS RECEIVED FROM THE FOLLOWING PUBLISHERS:—

J. Bale, Sons and Danielsson, Ltd.: "Scheme for Dealing with Tuberculous Persons." By D. Barty King, M.A., M.D. 5s. net.

J. and A. Churchill: "Malay Poisons and Charm Cures." By J. D. Gimlette, M.R.C.S., L.R.C.P. 3s. 6d. net.

T. Werner Laurie, Ltd.: "Painless Child-birth in Twilight Sleep." By Hanna Rion. 6s. net.

The Scientific Press, Ltd: "Children from Two to Five, their Care and Management." By Edith L. Maynard. 4d. net.

Rates of Subscription: Twelve months, 6/6; Six months, 4/-; Three months, 2/-, post free to any address in the United Kingdom. Abroad, Twelve months, 8/8; Six months, 5/-; Three months, 2/6, post free.—Address The Subscription Dept., "The Hospital," The Hospital Building, 28/29 Southampton Street, Strand, London, W.C.

**Twenty-Sixth Year of Publication.** Over 1000 pages. In Scarlet Cloth.

Price **10/6** net. ***Now Ready.*** By post, **10/11.**

### BURDETT'S
### HOSPITALS and CHARITIES, 1915.

The Year Book of Philanthropy and Hospital Annual.

**Edited by Sir HENRY BURDETT, K.C.B., K.C.V.O.**

"The Ideal of what a work of reference ought to be."—*The Times.*

**The volume for 1915 is the 26th Issue of "Hospitals and Charities," and contains SPECIALLY WRITTEN CHAPTERS on :—**

The Volume of Charity, 1913. The Future of the Hospitals. The King's Fund and the League of Mercy. The Nursing Department in 1913 and its Cost. Hospital Sunday. Hospital Saturday. Missions. Orphanages. The Blind. The Deaf and Dumb. Hospital Finance—Income, 1913; Expenditure, 1913. The United States, Canada, Australasia, and India. Convalescent Institutions. Hospital Construction, 1914.

**ALL WITH CAREFULLY ANALYSED FIGURES, AND A DIRECTORY OF**

Medical Universities, Colleges, and Schools.—London Hospitals.—London Dispensaries.—Metropolitan Asylums Board Hospitals.—Poor-Law Infirmaries in London and the Provinces.—Provincial Hospitals.—Provincial Infectious Hospitals.—Provincial Dispensaries.—Scotch Hospitals.—Scotch Dispensaries.—Irish Hospitals.—Lunacy Board and Lunatic Asylums: Indian, British, and Colonial.—Military and Naval Hospitals.—Convalescent Homes.—Sanatoria for Consumption.—Retreats for Inebriates.—Private Asylums.—Nursing Institutions.—Indian, Colonial, and American Hospitals.—Hospitals for Insane in the United States of America.—Administrative and Collective Societies.—Missionary and Religious Societies.—Orphanages, Homes, and Charities.—Institutions for the Blind.—Institutions for the Deaf and Dumb.—London County Council Centres for Blind Children.—London and Provincial Institutions for the Deaf and Dumb.—London County Council Centres for Deaf and Dumb Children.—Hospitals and Homes for Incurables.—Establishments for the Relief of Epilepsy.—Vaccine Lymph Establishments.—Reformatory and Industrial Schools.

**Obtainable of all Booksellers.** **Publishers: The Scientific Press, Ltd., London.**

